# Persistence of *Sarcocystis neurona* and histopathology in horses with equine protozoal myeloencephalitis

**DOI:** 10.3389/fvets.2026.1787994

**Published:** 2026-04-15

**Authors:** Lauren Helber, Bettina Wagner, Caroline M. Leeth, Tanya LeRoith, Thomas E. Cecere, Kevin K. Lahmers, Frank M. Andrews, Alayna N. Hay, Stephen R. Werre, Amy L. Johnson, Carol K. Clark, Nicola Pusterla, Stephen M. Reed, David S. Lindsay, Sandra D. Taylor, Krista E. Estell, Martin Furr, Robert J. Mackay, Fabio Del Piero, Mariano Carossino, Kacey Pandaleon, Savannah Weatherford, Roger Ramirez-Barrios, Kurt Zimmerman, Sharon G. Witonsky

**Affiliations:** 1 Department of Large Animal Clinical Sciences, Virginia- Maryland College of Veterinary Medicine, Virginia Tech Blacksburg, Blacksburg, VA, United States; 2 Department of Population Medicine and Diagnostic Sciences, Cornell University, Ithaca, NY, United States; 3 Department of Animal and Poultry Sciences, Virginia Tech, Blacksburg, VA, United States; 4 Department of Biomedical Sciences and Pathobiology, Virginia- Maryland College of Veterinary Medicine, Virginia Tech, Blacksburg, VA, United States; 5 Equine Health Studies Program, Department of Veterinary Clinical Sciences, School of Veterinary Medicine, Louisiana State University, Baton Rouge, LA, United States; 6 Department of Population Health Sciences, Virginia- Maryland College of Veterinary Medicine, Virginia Tech, Blacksburg, VA, United States; 7 Department of Clinical Studies – New Bolton Center, University of Pennsylvania School of Veterinary Medicine, Kennett Square, PA, United States; 8 Peterson and Smith Equine Hospital, Ocala, FL, United States; 9 Department of Medicine and Epidemiology, School of Veterinary Medicine, University of California, Davis, Davis, CA, United States; 10 Rood and Riddle Equine Hospital, Lexington, KY, United States; 11 Department of Veterinary Clinical Sciences, College of Veterinary Medicine, Purdue University, West Lafayette, IN, United States; 12 Department of Large Animal Clinical Sciences, Marion duPont Scott Equine Medical Center, Virginia- Maryland College of Veterinary Medicine, Virginia Tech, Leesburg, VA, United States; 13 Department of Physiological Sciences, Center for Veterinary Health Sciences, Oklahoma State University, Stillwater, OK, United States; 14 Department of Large Animal Clinical Sciences, University of Florida, Gainesville, FL, United States; 15 Department of Pathobiological Sciences, School of Veterinary Medicine, Louisiana State University, Baton Rouge, LA, United States

**Keywords:** acute, anti-protozoal treatment, chronic, degenerative changes, EPM testing, *Sarcocystis neurona* persistence

## Abstract

Currently, little is known about the exact role of *Sarcocystis neurona* immunopathology in equine protozoal myeloencephalitis (EPM), including the ability to persist after anti-protozoal treatment. The main objectives of this study were to determine whether *S. neurona* is present in the CNS in horses with EPM, including previously treated cases, and to evaluate the associated histopathology and immune response. For this study, control (*n* = 10) and horses with EPM (*n* = 9) were confirmed based on our inclusion criteria. Based on our preliminary data, we classified horses with EPM and clinical signs for >6 months as chronically affected (*n* = 5) while horses with clinical signs for <6 months were classified as acutely affected (*n* = 3). Histopathology changes between acute and chronic horses were identified. Identification of *S. neurona* was performed postmortem using PCR and IHC. Histopathology analysis with IHC identified *S. neurona* presence significantly more than PCR (*p* < 0.03) when directly compared. Histopathology analysis revealed that horses chronically affected with EPM had increased degenerative changes in their CNS compared to acutely affected horses when necropsy reports were compared. Our study indicates potential important histopathology differences between horses of varying clinical sign duration and the potential persistence of *S. neurona* after anti-protozoal treatment. Additional studies are warranted to further elucidate the role of *S. neurona* in disease, including the use of “acute” and “chronic” as clinical definitions for EPM.

## Introduction

Equine protozoal myeloencephalitis (EPM) is a devastating neurologic disease, predominantly caused by the protozoan *Sarcocystis neurona* (*S. neurona*) (1). The North American opossum (*Didelphis virginiana*) is a known definitive host of *S. neurona*, which is excreted through the feces (2). Horses are exposed to the protozoan through drinking water or eating food contaminated with opossum feces. Clinical signs of neurologic disease develop when the protozoa localize in the central nervous system (CNS) of the horse after ingestion. Horses are typically considered either dead-end or aberrant hosts, and not part of the organism’s normal lifecycle (3). Common clinical signs of EPM include facial nerve paralysis, lameness, ataxia, weakness, and muscle atrophy. Many of these signs can affect gait and could lead to recumbency. Most horses exposed to *S. neurona* do not develop clinical signs and less than 1% of horses develop clinical disease. Since most horses can mount a protective immune response and clear the protozoa before significant clinical signs occur, testing for antibodies in the serum alone is not definitive for diagnosing EPM (4). Therefore, a definitive antemortem diagnosis of EPM can be challenging (5). In horses euthanized due to their poor prognosis, a post-mortem examination is ideally conducted. The identification of *S. neurona* and associated histopathology changes is most conclusive in the confirmation of disease. While *S. neurona* can produce gross lesions within the CNS, lesions are not always present at the macroscopic or even microscopic level. Therefore, methods such as immunohistochemistry (IHC) and polymerase chain reaction (PCR) may be used to identify *S. neurona* to obtain a definitive post-mortem diagnosis (6, 7).

There are few published studies elucidating the disease progression, including the advancement of clinical disease, progression of histopathology changes, and the ability for *S. neurona* to persist after anti-protozoal treatment. Boy et al. (8) found that 30/53 (56%) of horses with EPM that had previously been treated with anti-protozoal treatment had *S. neurona* confirmed in the CNS (8). In comparison, Hay et al. (9) demonstrated that *S. neurona* can persist and replicate in an immunodeficient interferon-gamma (IFN- γ ) KO mouse model after anti-protozoal medication was suspended (9). Mice developed recurrent neurological signs and had identifiable *S. neurona* asexual stages in their CNS even though the mice were not re-exposed to the protozoa (9). Due to the limited studies showing *S. neurona* persistence after anti-protozoal treatment, further studies are warranted to identify the mechanism of *S. neurona* persistence after anti-protozoal treatment.

There have also been few published studies that have shown significant differences in the immune response between horses with EPM and control horses. Some studies have shown significant upregulation of IFN- γ and IL-12 in the serum of horses with EPM (10), while other studies have shown upregulation of IL-8, IL-10, TNF- α , and IFN- γ , in EPM-positive tissue though not significantly (11). Therefore, an investigation into potential cytokine and chemokine differences between horses with EPM and control horses is warranted.

The main objectives of our study were to identify whether *S. neurona* was present in the CNS of horses with EPM, particularly those horses previously treated for EPM, and to define the histopathology changes and the cytokine and chemokine response of horses in early and later stages of disease. Cytokines and chemokines were measured and documented in the serum and cerebrospinal fluid (CSF) of horses with EPM and control horses. IHC and PCR were also utilized to determine the presence or absence of *S. neurona* in horses with EPM and control horses, previously applied for postmortem diagnosis (6, 7, 12). Finally, the authors compared histopathology changes between acutely affected and chronically affected horses with EPM to identify any significant histopathology changes between the two groups.

## Materials and methods

### Horse selection and case definition

Horses were classified as having EPM if the horse had neurologic signs consistent with EPM and had a serum: CSF titer ratio of ≤100 based on a SnSAG 2/4/3 enzyme-linked immunosorbent assay (ELISA) (Equine Diagnostic Solutions; EDS, USA) (13). Horses with EPM were graded for severity of ataxia using the modified Mayhew scale, duration of clinical signs and treatment(s), and any other neurologic abnormalities were noted (14). Horses were considered to be a relapsed EPM horses if the horse had been seen by a veterinarian, treated for EPM with anti-protozoal medication, responded to treatment and then subsequently developed neurologic signs consistent with EPM at any time. Diagnostic testing was performed at the discretion of the veterinarian attending to the horse. Any horses not meeting our case definitions were excluded from the study.

Horses with no neurologic signs and no known history or evidence of local or systemic inflammatory disease (which could increase chemokine and cytokine concentrations) were designated as control horses. There were a total of 10 control horses that met our case definition. Control horses had a serum: CSF ratio of >100 on SnSAG 2/4/3 ELISA (EDS, USA), which demonstrated that they had been exposed to the protozoa previously but did not have clinical disease. All horses were donated for this study by the owners. Horses were euthanized with an intravenous administration of minimally 1 mL/10 lb. pentobarbital in accordance with euthanasia protocols approved for horses by the American Veterinary Medical Association (AVMA) and the Institutional Animal Care and Use Committee (IACUC).

### Tissue sample collection

Blood was collected from each horse via jugular venipuncture prior to euthanasia and sera was harvested after centrifugation. CSF was collected immediately after euthanasia from the cerebellomedullary cistern via an atlanto-occipital approach. Serum and CSF samples were submitted to Equine Diagnostic Solutions (Lexington, Kentucky, USA) for *S. neurona* SnSAG 2/4/3 ELISA testing (13). The lab was blinded to the status of the horse for the serum and CSF testing. The brain and spinal cord were removed by a board-certified pathologist at the time of necropsy. From each horse with EPM, a minimum of 3 separate 0.5–1 cm tissue sections were collected from each region of the spinal cord (i.e., cerebrum, brainstem, cervical (C1–C7) thoracic, lumbar, and sacral regions) that correlated with clinical signs and frozen at −80 °C in optimal cutting temperature (OCT) compound. Sections were taken at least 4–5 mm apart depending how large the spinal cord was. In addition, a 3mm^3^ portion of the tissue was snap-frozen in liquid nitrogen. Tissue samples adjacent to the samples collected for freezing were fixed in 10% formalin for histological analysis by the pathologists. Control horses had samples collected from the cerebrum, brainstem, cervical (C1–C7), thoracic, lumbar, and sacral regions. Three samples from each section of their spinal cord (ex., 3 samples from C1, 3 samples from C2, etc.) from each control horse were removed and frozen at −80 °C in OCT. In addition, a 3mm^3^ portion of the tissue was snap-frozen in liquid nitrogen. Tissue samples adjacent to the samples collected for freezing were fixed in 10% formalin for histological analysis by the pathologists.

### Histopathology examination, postmortem examination, and scoring

Horses that were euthanized all had post-mortem examinations to define their respective group (control, EPM). Sections were processed for routine hematoxylin and eosin (H&E) staining (15) and reviewed by a board-certified veterinary pathologist. The pathologist examined all sections for any histopathology changes in both control and horses with EPM. They looked for lesions consistent with EPM or other neurologic diseases. In the necropsy reports, the pathologists included any degenerative changes that were identified in the H&E slides from the horses with EPM and control horses. The presence of *S. neurona* organisms via IHC or PCR was not included as part of the inclusion criteria; only evidence or absence of gross or microscopic lesions consistent with EPM was considered for inclusion. The pathologist conducting the initial postmortem examination was not blinded to the horse’s disease status or putative diagnosis.

Two board-certified veterinary anatomic pathologists [MC and FD, Louisiana State University (LSU)], independently from the pathologists that collected the specimens (TL and TC) analyzed the histological specimens and scored the histopathology changes based on the criteria described. Based on the initial histopathology findings, the parameters analyzed were gliosis, axonal degeneration, neuronal degeneration, and inflammation. Each parameter was determined based on a scale of 0–3, 0 = normal, 1 = mild, 2 = moderate, and 3 = severe. Since some horses had more slides than others, the total score for the horses’ slides was averaged for each histopathology change.

### DNA isolation

DNA was extracted from the tissue using the Bioline Isolate II genomic DNA extraction kit, following the manufacturer’s instructions (16). DNA quality and concentration were evaluated by placing 1 μL of pure DNA elution concentration on a nanodrop-One^c^ machine at a double-stranded DNA setting to measure the concentration of the DNA from each sample that was extracted at room temperature (RT). DNA concentrations (ng/μL) were measured at a wavelength of 260 nm (17). The 260/280 range for the DNA was measured at ~1.8.

### Polymerase chain reaction (PCR)

A 1,100 bp fragment of the *S. neurona* DNA was amplified using primers JNB 33 (5′CGAACAGAGATGAGGAAAAT-3′) and JNB 54 (5′GTTGTGGTGTTGCGTGAGTC-3′) (18–20), using the Promega GoTaq Green Master (Promega, Wisconsin, USA). PCR conditions were: 94 °C for 3 min; 35 cycles of (94 °C for 30s, 56 °C for 60s, and 72 °C for 60s); and 72 °C for 10 min. PCR products were resolved on a 1% agarose gel. Positive and negative controls were used in every PCR.

### Immunohistochemical (IHC) staining

Slides of the cerebrum, brainstem, cervical, and lumbar sections of the horses with EPM and control horses were selected for immunohistochemical staining (IHC) (21). Tissues were cut from formalin-fixed, paraffin-embedded sections onto glass slides. Slides were washed at RT in xylene twice for 10 min (RT), 100% ethanol for 10 min (RT), 95% ethanol for 10 min (RT), 70% ethanol for 5 min (RT), 50% ethanol for 5 min (RT), and 1% PBS/BSA [VWR, USA; 0903-5G] for 5 min (RT). The slides were then washed in 3% hydrogen peroxide in 100% methanol solution (RT). Slides were then washed in 1% PBS/BSA twice for 5 min (RT). An immunopen [Millipore, Germany; 402176] was then used to draw around the sections, and a blocking solution containing goat serum was used to reduce non-specific binding from a Vector Laboratory kit [Vectastain ABC kit peroxidase, USA; PK-4001] for 20 min (RT). Then a 1:500 dilution of rabbit sera anti-*S. neurona* antibodies (21) were added and incubated at RT for 1 h. Negative controls received standard PBS [Biowhittaker, USA; 17-517Q] on the slides with no *S. neurona* antibody for both horses with EPM and control horse slides. Slides were then washed in 1% PBS/BSA 2 times for 5 min (RT). Then biotinylated antibody rabbit Ig [Goat anti-rabbit IgG (H + L); Vectastain] at RT (1 drop in 10 mL of 1% PBS/BSA) was dropped onto the sections and incubated for 30 min (RT). Slides were then washed 2 times in 1% PBS/BSA for 5 min (RT). Slides were next incubated with an ABC reagent for 30 min (RT) and then washed 2 times in 1% PBS/BSA for 5 min (RT). Fifteen mL of 0.05 M Tris [Fischer, USA; BP152-500] saline, DAB tablet [Sigma Aldrich, USA; D5905-50TAB], and 3.5 μL of 30% hydrogen peroxide [VWR; BDH7690-1] were placed on the slides for 1 min and washed off (RT). The slides were run under tap water and placed in hematoxylin [Thermo Scientific, USA; 7,211] for 5 min (RT) and then counterstained in 10% Scott’s tap water [Sigma Aldrich; 55,134] solution for 30 s (RT). The sections were dehydrated in 50% ethanol for 5 min (RT), 70% ethanol for 5 min (RT), 95% ethanol for 5 min (RT), 100% ethanol for 5 min (RT), and xylene for 10 min (RT) (6). Slides were then mounted with Permount and examined for *S. neurona* positive staining with a compound Olympus microscope. Negative and positive control slides were used to ensure the identification of *S. neurona.*

### Cytokine and chemokine assay

Cytokines and chemokines were analyzed in the serum and CSF in two quantitative fluorescent bead-based assays. One assay uses a multiplex-based assay for TNF-α, IL-1β, CCL2, CCL3, CCL11, and CCL5 using monoclonal antibodies for equine chemokines (22, 23). A separate cytokine 5-plex fluorescent bead-based assay was used to measure IFN-α, IL-4, IFN- γ, IL-10, and IL-17 (24). Cytokine and chemokine analyses were performed at the Animal Health Diagnostic Center at Cornell University.

### Statistical analysis

A distribution test was applied to determine if the cytokine and chemokine data between control horses, acutely affected horses, and chronically affected horses with EPM were normally distributed. Normally distributed data were summarized as means± standard deviation, and the groups were compared using an analysis of variance (ANOVA) test. Data that were not normally distributed were examined using a Wilcoxon rank sum/ Kruskal-Wallis non-parametric test. *p*-values were adjusted for multiple comparisons using Tukey’s procedure (ANOVA) or Dunn method for joint ranking (Kruskal–Wallis). This same statistical procedure was run for the comparison of the histopathology scores for the control horses, acutely affected, and chronically affected EPM horses. For the comparison of the ability of IHC vs. PCR to detect *S. neurona* presence, a McNemar’s chi-squared test was used. Significance was established at *p* < 0.05. All analyses were performed using JMP Pro Version 16 (Cary, NC., USA).

## Results

### Horse selection and case definitions

A total of (*n* = 9) EPM and (*n* = 10) control horses met the criteria for inclusion in the study. Horses with clinical signs of EPM and control horses were recruited and donated to the Virginia Maryland College of Veterinary Medicine. Eight horses had serum: CSF ratios of <100, and one horse had a serum: CSF ratio of = 100. Even though one horse had a serum: CSF ratio = 100, the horse had accompanying clinical signs consistent with EPM, and evidence of EPM lesions in post-mortem examination. Therefore, this horse was included as a horse with EPM. Horses of varying breeds, ages, and sexes were included, and a brief history was noted. Breeds included quarter horses (*n* = 4), thoroughbreds (*n* = 8), Tennessee walking horses (*n* = 2), warmbloods (*n* = 3), and mixed breeds (*n* = 2). The horses ranged in age from 6 to 29 years. The mean age for horses affected by EPM was 17.4 years, and the control horses’ mean age was 16.4 years. The majority of the horses were geldings, 88.8% (8/9), and 88.8% (8/9) of the horses were over 10 years of age. Forty-four percent (4/9) of the horses had previously been treated with anti-protozoal medications at least twice for EPM and had relapsed at least once. All four of the horses that had relapsed were geldings.

Based on our preliminary data, as horses were included in the study, horses with clinical signs of >6 months were classified as chronically affected, and horses with clinical signs of <6 months were considered acutely affected. Three of the five horses with chronic EPM had been treated at least twice for EPM and relapsed at least once. Only 1/3 horses with acute EPM had previously been treated for EPM. Although the one horse with acute EPM had previously been treated with anti-protozoal medications, the time from onset of clinical signs and euthanasia was still less than 6 months, and therefore, the horse was still considered an acutely affected horse. One horse was donated and presented with an unknown duration of clinical signs and was not included in the comparison.

Ten horses met our case definition of controls, as they had no previous or current history of neurologic disease. All 10 horses had serum: CSF ratios of >100 for antibodies against *S. neurona* in a SnSAG 2/4/3 ELISA test, indicating that they had been exposed to *S. neurona* but had not developed clinical disease.

### Neurologic examination results

The most common clinical signs/neurologic deficits of the horses with EPM were paresis associated with a weak tail pull, spasticity, as well as ataxia in the pelvic limbs in 4/9 (44.4%) (Table 1). Other clinical signs included asymmetric muscle atrophy of the pelvic limbs (2/9), irritability (1/9), and blindness (1/9). The horses with chronic EPM (n = 5) displayed thoracic or pelvic limb proprioceptive deficits (3/5), ataxia (3/5), spasticity of the thoracic or pelvic limbs (3/5), weak tail pull (paresis) (2/5), irritability (1/5), and weakness of the pelvic limbs (1/5). Horses acutely affected with EPM had thoracic or pelvic limb delayed proprioception (2/3), generalized ataxia (3/3), spasticity of their thoracic or pelvic limb (1/3), and paresis on tail pull (1/2). One acutely affected horse was too weak to perform a tail pull. A summary of clinical signs between acute and chronic horses is presented in Table 1.

**Table 1:** Clinical signs between acutely affected and chronically affected EPM horses.

Clinical signs	Acutely affected	Chronically affected
Ataxia	2/3	3/5
Hind or front-end delay	2/3	3/5
Spasticity in hind or front end	1/3	3/5
Weak tail pull	1/3	2/5
Asymmetric muscle condition	1/3	1/5

### Histopathology differences between acutely and chronically affected horses

**Figure 1:**
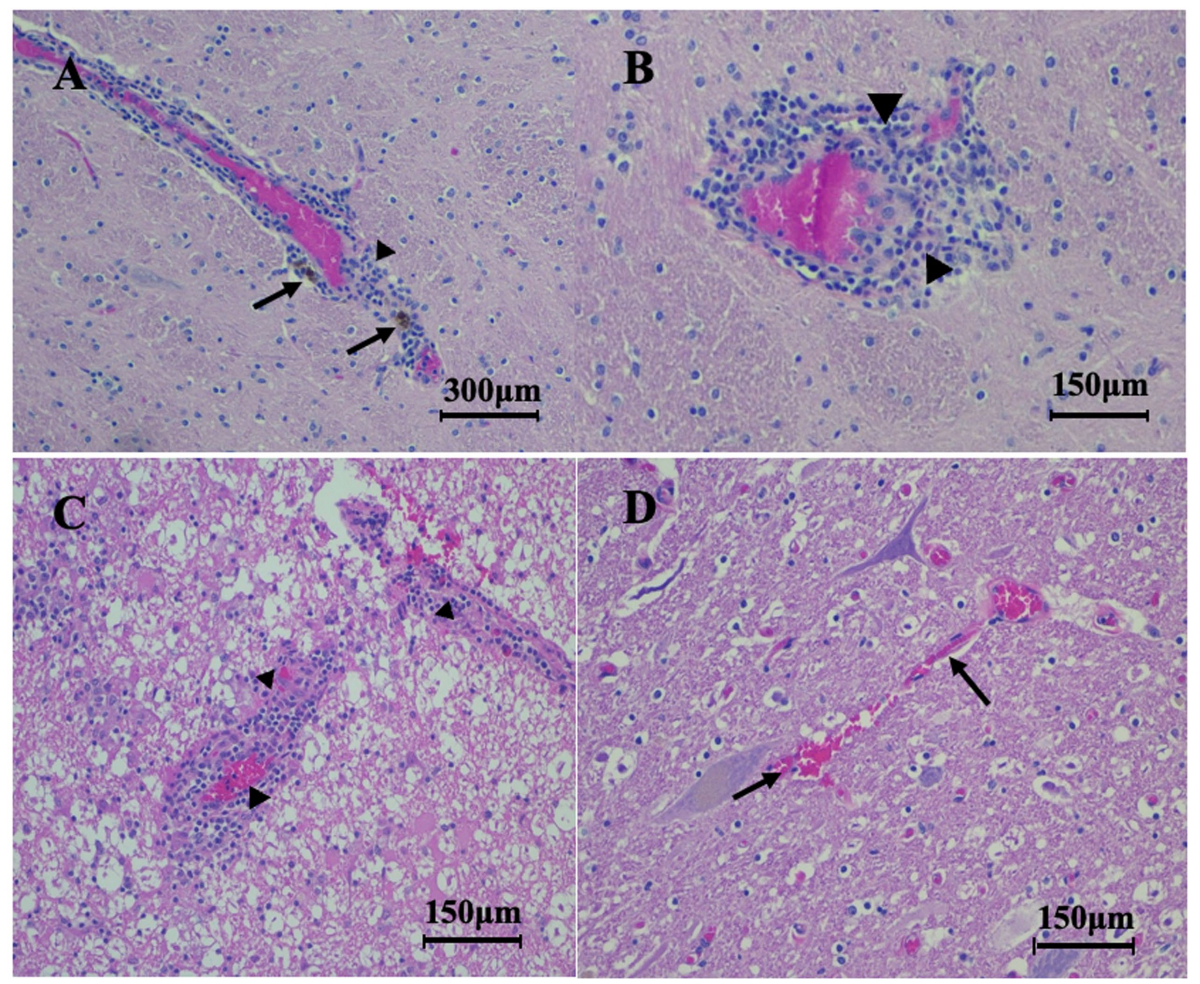
H&E-stained histopathology sections from acutely affected, chronically affected EPM, and control horses. **(A)** Inflammatory cells and perivascular cuffing (arrowhead) and signs of hemorrhaging (arrows) from the thalamus of a horse chronically affected with EPM (100x). **(B)** Perivascular cuffing (arrowhead) from a horse chronically by EPM (200x). **(C)** Perivascular cuffing and inflammatory cells (arrowheads) from the spinal cord of a horse acutely affected by EPM (200x). **(D)** A normal blood vessel without perivascular cuffing (arrows) from the lumbar spine of a control horse (200x).

Based on our case definitions, there were three acute and five chronic horses with EPM. The most common histopathology changes in horses with EPM included lymphocytic infiltration, eosinophilic inflammation, gliosis, perivascular cuffing, and degenerative changes. Degenerative changes included scarring of neural tissue, neuron degeneration, axonal degeneration, and spheroid formation. Of the five horses chronically affected by EPM, 5/5 (100%) horses had axonal degeneration or swelling, while 4/5 (80%) horses had swollen myelin sheaths and lymphocyte infiltration (Figure 1). Gliosis was identifiable in 3/5 horses (60%) (Table 2).

**Table 2:** Demonstration of, and comparison of, degenerative and other histopathologic changes between chronically and acutely infected EPM horses.

Inflammatory changes	Acute	Chronic
Neuronal necrosis	2/3	0/5
Hemorrhage	3/3	0/5
Lymphocyte infiltration	3/3	4/5
Grey matter inflammation	2/3	0/5

Degenerative changes	Acute	Chronic
Axonal degeneration/swelling	2/3	5/5
Swollen/dilated myelin sheaths	0/3	4/5
Gliosis	0/3	3/5

These data reflect the comparative histopathological changes between horses from the necropsy reports.

One of three horses acutely affected by EPM had both gross lesions and histopathology lesions, while the other two horses acutely affected by EPM had only histopathology lesions in their CNS. The spinal cord tissue samples collected from healthy control horses were unremarkable and had no histopathology consistent with EPM. All (3/3) (100%) of the horses acutely affected by EPM had lymphocytic infiltrates (Figure 1) in their examined spinal cord sections, while 2/3 (66.6%) horses acutely affected by EPM had grey matter inflammation as well as neuronal necrosis (Table 2).

**Figure 2:**
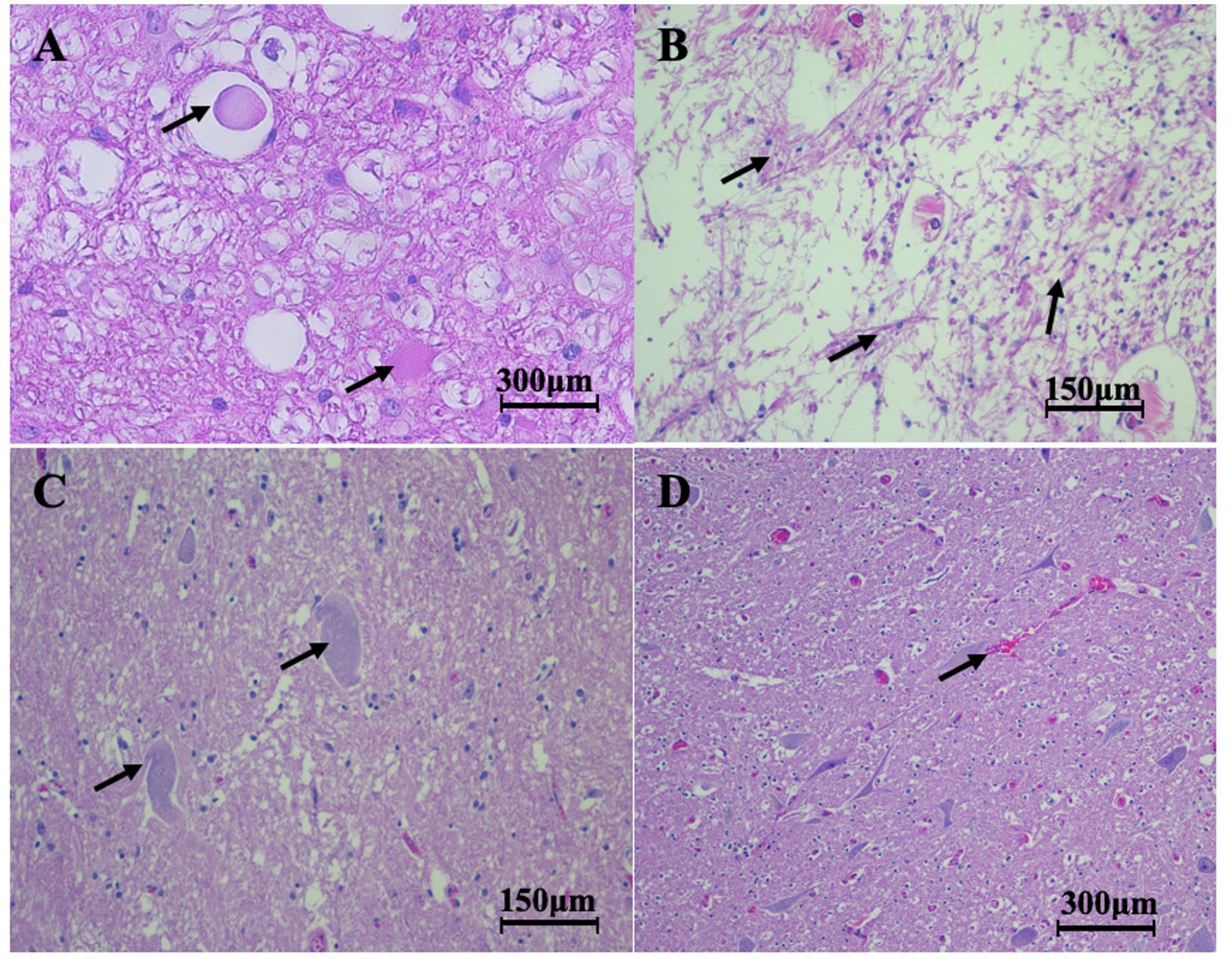
H&E-stained histopathology sections from acutely affected, chronically affected, and control horses. **(A)** Spheroid formation (arrows) in the spinal cord of a horse chronically affected with EPM (400x). **(B)** Scarring in the spinal cord (arrows) of a horse chronically affected with EPM (200x). **(C)** Neuron degeneration (arrows) in the lumbar section of a horse chronically affected by EPM (200x). **(D)** A normal lumbar section from a control horse (100x).

Overall, the horses acutely affected with EPM had fewer histopathological degenerative changes (Figure 2) compared to horses chronically affected by EPM when their necropsy reports were examined. The combined results of horses acutely and chronically affected by EPM are summarized in Table 2.

**Figure 3:**
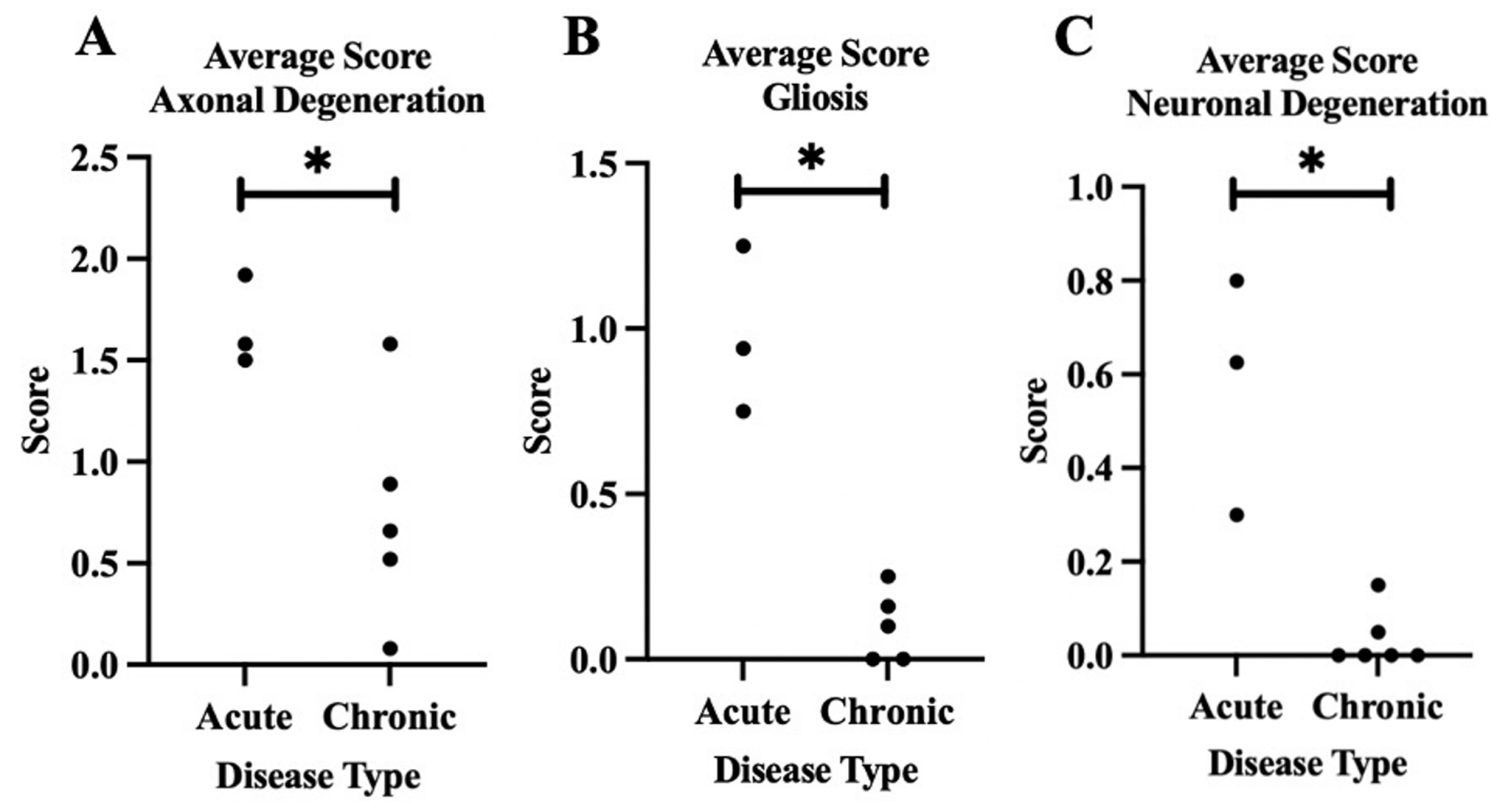
The averages of horses acutely and chronically affected by EPM (0 = normal, I = mild, 2 = moderate, 3 = severe). These data reflect the averages for the quantitative histopathologic scoring from LSU. **(A)** Comparison of axonal degeneration in horses acutely and chronically affected by EPM. **(B)** Comparison of gliosis in horses acutely and chronically affected by EРМ. **(C)** Comparison of neuronal degeneration in horses acutely and chronically affected by EPM. *Denotes *p* < 0.05.

**Figure 4:**
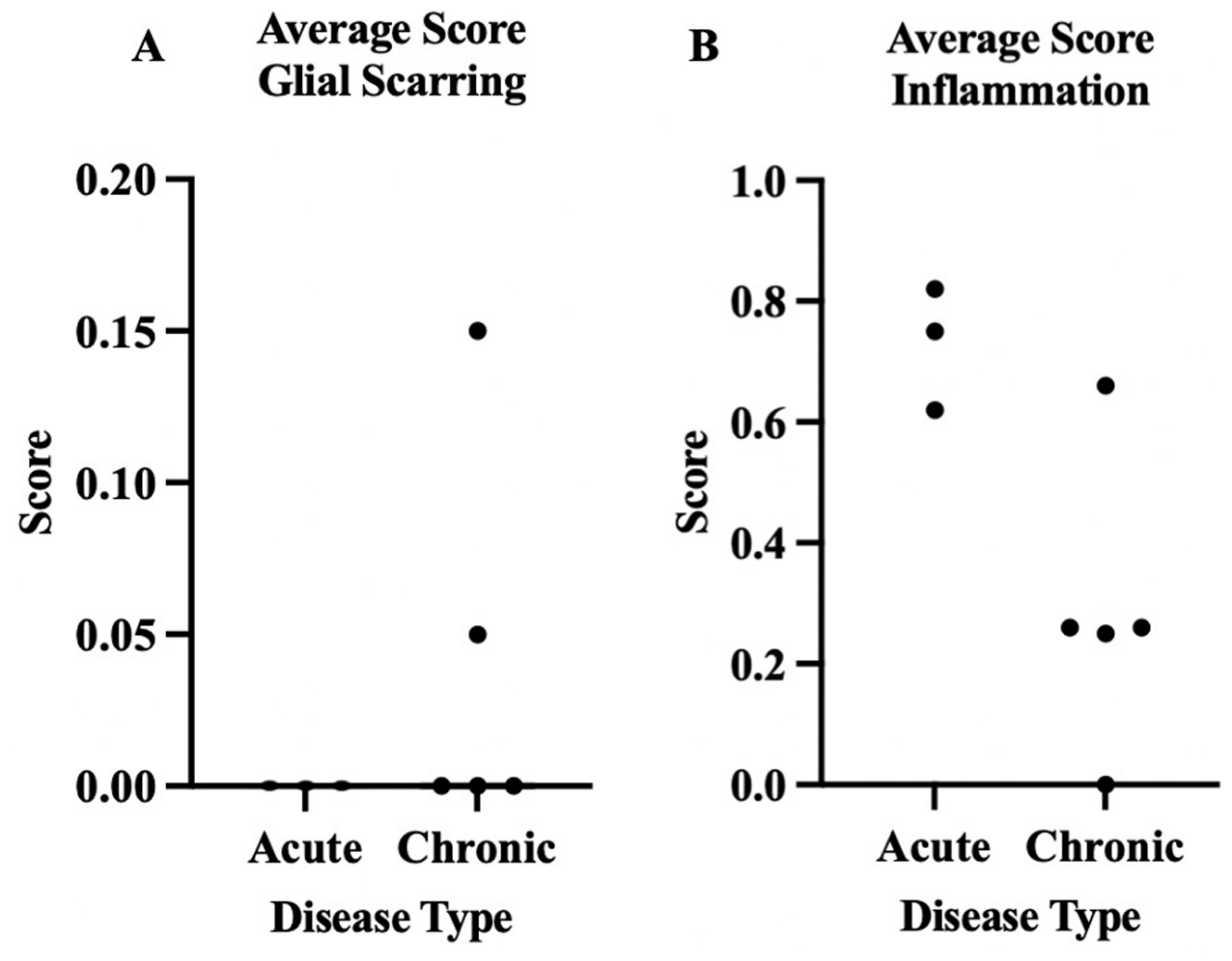
The averages of horses acutely and chronically affected by EPM (0 = normal, 1 = mild, 2 = moderate, 3 = severe). These data reflect the averages for the quantitative histopathologic scoring from LSU. **(A)** Comparison of glial scarring between horses acutely and chronically affected by EPM. **(B)** Comparison of inflammation in horses acutely and chronically affected by EPM. *Denotes *p* < 0.05.

For our statistical analysis, we utilized the three acute and five chronic horses with EPM. Based on the lesion scoring done by LSU, we found significant differences (*p* < 0.03) between horses acutely and chronically affected by EPM for gliosis, axonal degeneration, and neuronal degeneration. There were no significant differences between the two groups for inflammation and glial scarring (Figures 3, 4).

### *Sarcocystis neurona* PCR testing results

Four out of nine horses (44.4%) with EPM tested positive on tissue samples for *S. neurona* presence using PCR. Of the 10 control horses, 3 had available tissue sections for PCR, and all 3 control horses were negative for *S. neurona* presence by PCR testing.

### *Sarcocystis neurona* IHC results

**Figure 5:**
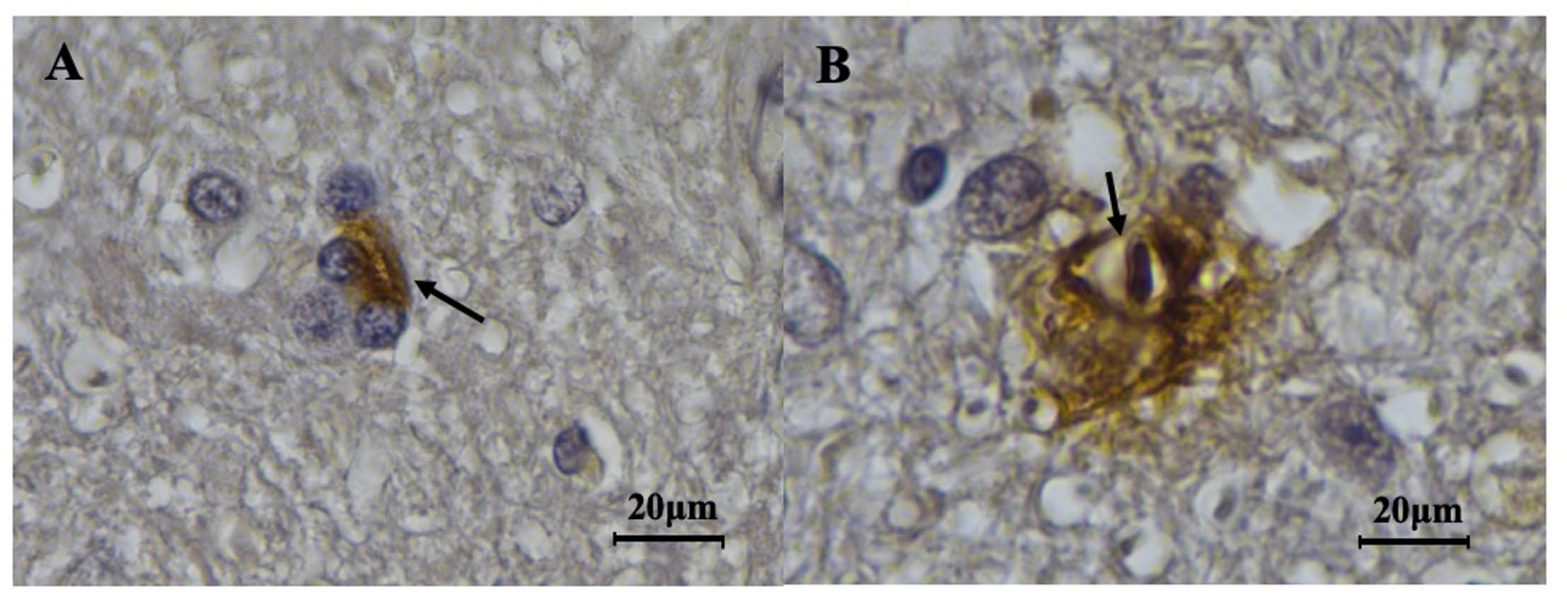
Presence of *S. neurona* merozoites in acutely and chronically affected EPM horse CNS. **(A)** Inflammatory cells surrounding an *S. neurona* merozoite (arrow) from a horse chronically affected by ЕРМ (1,000x). **(B)** A *S. neurona* merozoite (arrow) from an acutely affected horse (1,000x).

All 9 horses with EPM were positive for *S. neurona* presence on at least one slide by immunohistochemical (IHC) detection (Figure 5). Samples were available from 8 of 10 control horses, and all eight horses were negative for *S. neurona* presence using IHC. Two control horses did not have sections available for IHC or PCR testing but were included as controls based on our case definition criteria.

All horses with EPM were positive for *S. neurona* presence, either by IHC detection or by both IHC and PCR detection methods. Four of 9 (44.4%) horses with EPM tested positive for *S. neurona* presence with PCR testing and IHC testing, whereas all nine horses with EPM were positive for *S. neurona* presence using IHC.

The three control horses that were negative by PCR were also negative by IHC for *S. neurona* presence. Out of the 10 control horses, the eight that were available for testing were negative for *S. neurona* presence in either PCR or IHC.

There was a statistically significant difference in the ability of IHC to detect *S. neurona* vs. PCR (*p* < 0.03).

### Cytokine analysis

Of the 19 total horses that were used in this study, 18 horses had serum and CSF available for cytokine and chemokine analysis. The cytokines were analyzed based on horses affected by EPM (*n* = 8) and control horses (*n* = 10) for comparison. Then horses with EPM were further split into acutely affected (*n* = 3) and chronically affected (*n* = 4) for comparison.

Some of the chemokines and cytokines that are predicted or known to be important in protection and had a trend toward significance and could be biologically significant are presented in Table 3. The cytokines chosen are cytokines associated with the immune response in *S. neurona* infection (25). There were no statistically significant differences in cytokines and chemokines between horses affected with EPM and control horses.

**Table 3:** Partial chemokine and cytokine concentrations (mean ± SD ) in serum and CSF of EPM and control horses.

Cytokine/Chemokine	Horse	Avg serum	CSF	Serum *p*-value	CSF *p*-value
IL-17A	EPM	14.33 ± 4.4 (U/mL)	1.5 ± 0.2 (U/mL)	0.13	0.4
	Control	7 ± 3.62 (U/mL)	1.3 ± 0.2 (U/mL)		
IFN- γ	EPM	11.5 ± 4.7 (U/mL)	3 ± 0.4 (U/mL)	0.09	0.35
	Control	11.89 ± 3.8 (U/mL)	4.1 ± 0.3 (U/mL)		
IL-1 β	EPM	194.6 ± 140.8 (pg/mL)	61.63 ± 47.2 (pg/mL)	0.42	0.27
	Control	0 ± 104.9 (pg/mL)	0 ± 42.2 (pg/mL)		
TNF- α	EPM	0 ± 25.1 (pg/mL)	170 ± 68.3 (pg/mL)	0.83	0.26
	Control	27.88 ± 18.73 (pg/mL)	91.6 ± 61.1 (pg/mL)		

### Persistence of *Sarcocystis neurona*

In our study, we wanted to observe if there was any sex bias between horses that had relapsed after previously being treated for EPM. The four horses that had been previously treated for EPM were all geldings, and all four had *S. neurona* present in their CNS after anti-protozoal treatment.

## Discussion

From this study, we have identified: (1) *S. neurona* was present in all horses with EPM, including the four horses that received anti-protozoal treatment, and (2) identified the differences in histopathology lesions between the acutely and chronically affected horses with EPM. In this small study, IHC was more accurate in identifying *S. neurona* in all spinal cord tissue sections compared to PCR, which detected *S. neurona* in 44.4% of the horses with EPM. In PCR testing, DNA was extracted from only 25 mg of tissue, and if *S. neurona* was not present in the tissue that was extracted, the likelihood of getting a positive *S. neurona* result was low. In IHC testing, an entire cross-section of a tissue sample on the slide was tested, which provided a larger sample area to detect *S. neurona*. The industry standard for 40 cycles of PCR was not used as described in (26). Instead, 35 cycles were used as described in (18, 19, 27). In these studies, 35 cycles of PCR were enough to allow for *S. neurona* DNA amplification, and the methods of the papers were, in our opinion, sufficient to accurately detect *S. neurona*. However, because of the 35 cycles vs. 40 cycle differences, there was a possibility that any *S. neurona* was present, but not enough replication cycles were performed to detect *S. neurona*. Also, comparing the use of conventional PCR versus qPCR, the latter of which gives a more sensitive and quantitative result, was not used. This could have also contributed to the failure to identify *S. neurona* in some of the tissue sections by PCR. Traditional PCR was used based on previous studies which we believed sufficient to detect *S. neurona* (18, 19). Positive controls and negative controls, as well as duplicate samples, were tested. In addition, retesting the CNS samples multiple times allowed the authors to rule out false positives and negatives on the PCR results, however, we did not incorporate an internal control to rule out inhibition, which could have affected our PCR results.

It was the combination of the PCR and IHC assays that provided the data supporting our second significant finding that *S. neurona* was present in all horses with EPM, including horses previously treated with anti-protozoal medication. Although the sample size is small, our data support that *S. neurona* can persist after anti-protozoal treatment. It is possible that the horses that had previously been treated for *S. neurona* were re-exposed to the protozoa and then subsequently developed clinical disease. However, Hay’s previous study done in mouse models suggests that the protozoa have the ability to persist after treatment and re-infect the host without being exposed to the protozoa again (9). Future studies are warranted to further define the ability of *S. neurona* to persist after anti-protozoal treatment.

From this study, we identified that horses chronically affected by EPM had more degenerative histopathology changes in their CNS than the acutely affected horses with EPM when comparing their histopathology changes from their necropsy reports. The presence of these monocytic infiltrates in both the acutely and chronically affected horses with EPM may be due to the presence and response to *S. neurona*, whereas acutely affected horses with EPM had fewer degenerative changes. Monocytic infiltration was the most significant histopathology change in the horses acutely affected by EPM. However, when the endpoints of inflammation and degeneration were quantitatively scored by co-authors Carossino and DelPiero, the horses acutely affected by EPM had similar degenerative changes compared to the horses chronically affected by EPM. The horses acutely affected by EPM had significantly higher axonal degeneration, gliosis, and neuronal degeneration compared to the horses chronically affected by EPM. Horses chronically affected with EPM did have increased glial scarring, which is suggestive of degeneration; however, it was not significant compared to horses acutely affected by EPM. These results could be due to the smaller sample size.

The authors acknowledge the necropsy reports and the scoring analysis conflict. Compared to the quantitative analysis, necropsy reports are limited in the amount of information they report, while the quantitative scoring of the histopathology changes is more in-depth, which may have caused the conflicting results. We also only examined five different histopathology changes for our quantitative analysis as indicated by the LSU co-authors. The five histopathology changes were chosen to best indicate the inflammatory versus the degenerative changes for the analysis. However, since we did not quantify every histopathology change, this may have affected our findings. Histopathology sections are taken from many sections of the spinal cord, but it is impossible to take every section. Therefore, not all sections of the spinal cord can be analyzed. The acutely affected EPM horses could have had increased degenerative changes because of the acute inflammatory response associated with active infection. A few of the horses chronically affected with EPM had previously been treated for *S. neurona* infection and were relatively stable neurologically, so it is possible the horses that were treated had a chance to recover after initial infection, which could impact our findings.

Several studies have indicated the terminology of “acute” and “chronic” for the onset of clinical signs and histopathology findings (28–30). These studies reference the acute and subsequent chronic onset of clinical signs associated with EPM. Acute lesions are usually consistent with multifocal and random distribution foci of hemorrhaging, which was seen in our acutely affected EPM horses (31). Chronic lesions show discoloration that can range from pale to dark and were found in our chronically affected EPM horses (data not shown) (31). One chronic horse in our study had clinical signs of EPM intermittently over 10 years before being euthanized. With horses that have the potential for relapse after treatment, a <6-month acute distinction, and a >6-month chronic distinction may not be out of range. Because of the limited number of horses from this preliminary study, conclusions on the definition of “acute” and “chronic” cases of EPM histologically cannot be confirmed. However, the use of “acute” and “chronic” as a clinical definition may prompt future studies to further support our conclusions. Further studies to elucidate the role of *S. neurona* in degenerative changes in horses with EPM are warranted, including the use of “acute” and “chronic” as clinical definitions for horses with EPM.

It is interesting that all affected horses that had been treated with anti-protozoal medication and subsequently relapsed were male, suggesting a potential sex bias for EPM susceptibility. While only two studies have indicated potential EPM susceptibility via sex bias (8, 32), many other studies have not indicated a sex bias for EPM. This data may support future studies to elucidate if there is sex bias.

While previous studies have demonstrated that *S. neurona* can be detected in the spinal cord of horses’ post-mortem utilizing IHC (6) as well as PCR (7, 12), the sensitivity and specificity cutoff from these tests to diagnose *S. neurona* have not yet been determined. Our study reports that in this small group of horses, IHC was, in our case, more sensitive at detecting *S. neurona* compared to PCR testing. IHC was able to detect *S. neurona* in 9/9 horses with EPM, while PCR was only able to detect *S. neurona* in 4/9 horses with EPM. Although only three control horses were tested for *S. neurona* presence, the same horses tested negative for *S. neurona* in IHC staining. The control horses followed our case definitions which allow them to still be considered healthy controls even with few samples. Further studies using a larger population of horses are warranted to determine the sensitivity, specificity, and accuracy of IHC and PCR to detect *S. neurona.* The use of qPCR may be warranted to quantify the amount of *S. neurona* that is being replicated.

In addition to these differences in the sensitivity of IHC vs. PCR to detect *S. neurona*, another significant finding is the differences in the lesions in the horses acutely and chronically affected by EPM. In the absence of gross and histopathology lesions at necropsy, IHC and/or PCR results can aid in the diagnosis of EPM in horses that have compatible clinical signs with EPM. Typical lesions in horses affected by EPM can be focal or multifocal, and gross lesions are typically characterized by segmental areas of hemorrhage. Microscopically, areas of hemorrhage, necrosis, and perivascular cuffing are suggestive but not definitive for infection (5). In our study, pathologic examination of the horses with EPM indicated occasional gross lesions as well as evidence of microscopic lesions with lymphocytic infiltration, perivascular cuffing, hemorrhage, and necrosis. Further studies with a larger number of cases are indicated to identify if horses chronically affected by EPM have increased degenerative changes and deteriorate clinically, compared to horses acutely by EPM.

We sought to compare cytokine and chemokine responses between horses with EPM and control horses. We hypothesized we would be able to better elucidate the immune phenotype of the horses with EPM when we assessed their immune responses and pathology compared to the seropositive neurologically horses because they had been exposed but did not develop clinical disease. While there were no statistically significant differences in the cytokine or chemokine serum and CSF samples, the sample size was small, and the groups could not be divided into treated or untreated cohorts. In addition, it is predicted that because each horse was at a different stage of disease, immune response, and pathological progression, the horses’ immune response could have been differentially affected. This individual immune response would affect the serum and CSF cytokine concentration as well. Further studies are warranted to identify if there are significant differences between cytokine expression in horses with EPM and control horses.

The key results from this study are that the authors identified *S. neurona* in all horses affected by EPM, despite some horses having previously been treated with anti-protozoal medications. This raises awareness of the potential for persistent infection or new exposure causing disease. Additionally, in this small study, we identified that IHC was significantly more sensitive than conventional PCR to identify *S. neurona* in the CNS of horses with EPM. Finally, identifying those horses with chronic infection having more degenerative changes compared to those acutely affected has not previously been published. While it is unclear if the protozoa cause degenerative changes to the CNS, or if the changes are a result of the inflammatory response, this study indicates that the presence of *S. neurona* may contribute to increased overall degenerative changes in acutely and chronically affected horses. We believe these important histopathology changes in these horses with EPM and the potential persistence of *S. neurona* will allow researchers to further understand the pathophysiology of the disease and allow for further exploration into the mechanism of how *S. neurona* causes damage to the CNS in advanced cases. With this additional knowledge, we predict additional refined diagnostics and improved immune therapies will enhance our treatment and improve outcomes of these horses. Additional studies are warranted to further define the neuro- and immunopathology, including the mechanisms for *S. neurona* persistence as well as determining the accuracy of PCR and IHC for diagnosis.

## Data Availability

The datasets presented in this study can be found in online repositories. The names of the repository/repositories and accession number(s) can be found in the article/supplementary material.
